# Progress of arylacetamide deacetylase research in metabolic diseases

**DOI:** 10.3389/fonc.2025.1564419

**Published:** 2025-05-01

**Authors:** Liu Yang, Zhe-Zhen Liao, Li Ran, Xin-Hua Xiao

**Affiliations:** Department of Metabolism and Endocrinology, The First Affiliated Hospital, Hengyang Medical School, University of South China, Hengyang, Hunan, China

**Keywords:** AADAC, metabolic diseases, structure, drug metabolism, lipid metabolism, obesity, diabetes, cancer

## Abstract

Arylacetamide deacetylase (AADAC), a microsomal serine esterase belonging to the polygenic hydrolase family, is predominantly localized in the liver and intestine. It plays a significant role in drug metabolism, lipid metabolism, and the pathogenesis of various diseases. In the context of drug metabolism, AADAC is vital for ensuring the safety of ester-based drugs. Its substrate specificity for short-chain acyl groups, along with genetic polymorphisms among individuals and species, influences drug-related processes. Regarding lipid metabolism, The lipase activity of AADAC is involved in the hydrolysis of cholesterol and triglycerides, lipid mobilization, and the assembly of lipoproteins. The expression of AADAC is regulated by multiple factors. It is associated with metabolic disorders; for instance, its decreased expression in the liver during obesity may impact triglyceride metabolism, and it may also have an indirect role in diabetes. In cardiovascular diseases, AADAC holds potential as a diagnostic marker. Its role in cancer is heterogeneous, being downregulated in certain cancers while upregulated in others, such as pancreatic and ovarian cancers, where it acts to inhibit cancer progression. Within the nervous system, AADAC may influence neurotransmitter regulation and drug metabolism. Currently, research on AADAC agonists is limited, and the development of inhibitors presents challenges, underscoring the necessity for further investigation in this area.

## 1 Introduction

Arylacetamide deacetylase (AADAC), a microsomal serine esterase predominantly located in the liver and intestine, is a member of the polygenic hydrolase family. Its primary function involves catalyzing the hydrolysis of acetyl groups, thereby contributing to the metabolism and detoxification of various endogenous substances, such as cholesterol esters and triglycerides, as well as xenobiotics, including rifampicin and flutamide. In recent years, the increasing global prevalence of metabolic diseases has highlighted the distinctive role of AADAC in the regulation of lipid metabolism as a significant area of research interest.

Studies suggest that AADAC is intricately associated with the pathogenesis of metabolic disorders, including obesity, type 2 diabetes, and atherosclerosis, through its influence on fatty acid oxidation, very low-density lipoprotein (VLDL) assembly, and triglyceride homeostasis. For example, diminished hepatic AADAC expression in obese individuals may exacerbate lipid accumulation, while genetic polymorphisms of AADAC could contribute to interindividual variability in drug metabolism and disease phenotypes. Moreover, the expression of AADAC is modulated by transcription factors, microRNAs, and inflammatory cytokines, underscoring its intricate role in the maintenance of metabolic homeostasis. Despite this, the molecular mechanisms governing AADAC’s dual regulatory functions—such as its ability to either suppress or promote tumor progression—remain inadequately elucidated. Additionally, the development of specific AADAC agonists or inhibitors is hindered by challenges including interspecies differences and individual genetic variability. This review systematically consolidates recent advancements in AADAC research within the realm of metabolic diseases, investigates its potential as a therapeutic target, and critically examines current limitations. The objective is to provide a theoretical foundation and translational direction for the precise intervention of metabolic disorders.

## 2 Molecular structure and catalytic mechanism of AADAC

AADAC, a 45 kDa microsomal serine esterase from the polygenic hydrolase family, is mainly found in the liver and intestine. Since its purification from human liver in 1991, research has advanced (1). By 1994, its full-length cDNA was isolated, showing an active site similar to carboxylesterases, highlighting its unique classification (2). In 1997, Yamazaki mapped the AADAC gene to 3q21.3–q25.2, facilitating future functional studies (3).

**Figure 1:**
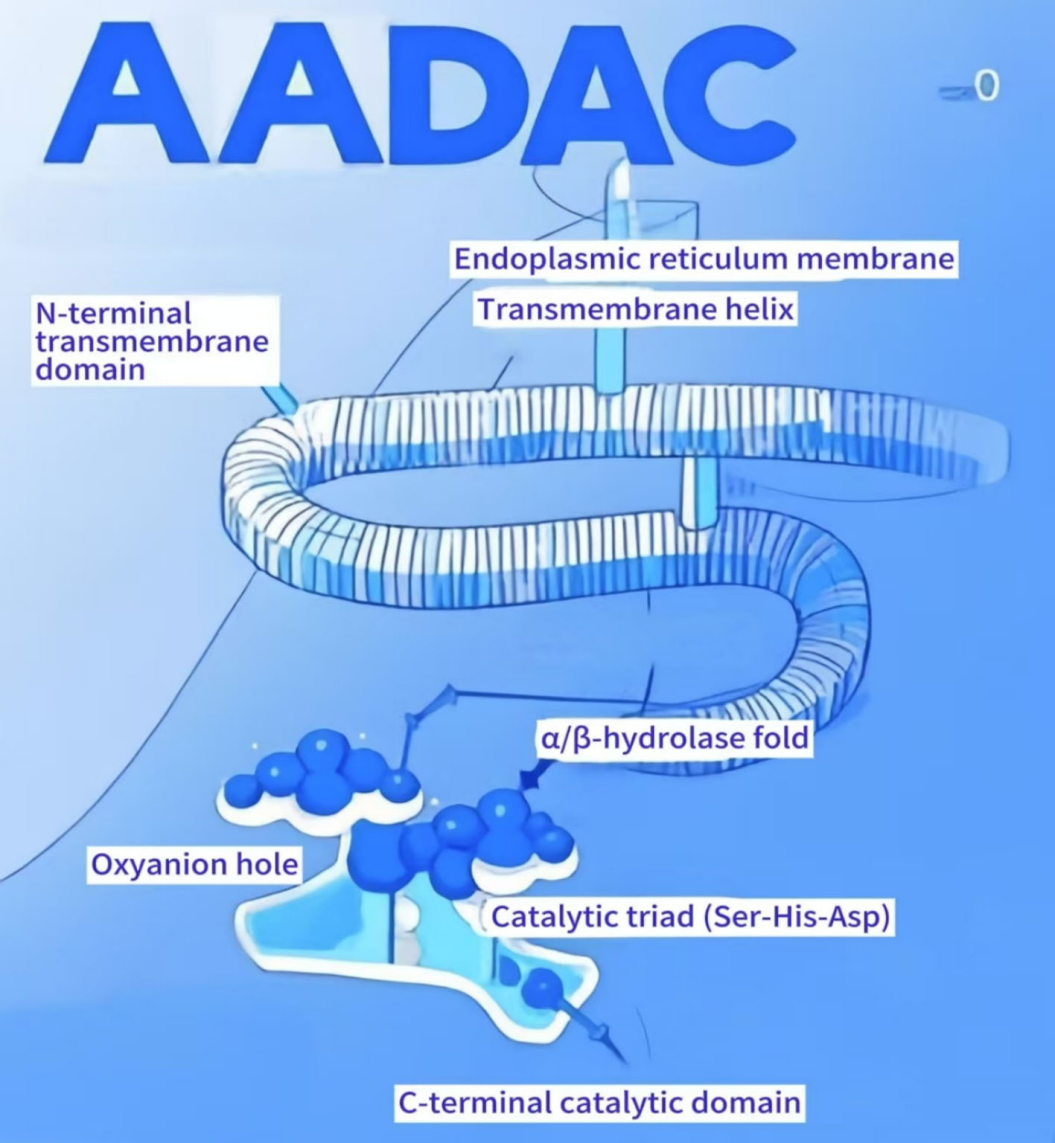
Schematic diagram of AADAC structure. AADAC comprises two principal domains: an N-terminal transmembrane domain anchored to the endoplasmic reticulum membrane via a hydrophobic α-helical segment, ensuring luminal orientation for lipid metabolism and xenobiotic detoxification, and a C-terminal catalytic domain adopting a canonical α/β-hydrolase fold. The catalytic core consists of eight parallel β-strands flanked by α-helices, forming a hydrophobic substrate-binding pocket. Embedded within this domain are the conserved catalytic triad (Ser217, His438, Asp367) and an oxyanion hole, critical for transition-state stabilization during hydrolysis. These structural elements collectively govern substrate specificity and catalytic activity.

As a type II membrane protein, the N-terminal transmembrane domain of AADAC is anchored to the endoplasmic reticulum (ER) membrane through a transmembrane helix, facilitating its localization on the luminal side of the ER. This positioning is critical for its involvement in lipid metabolism and drug detoxification processes (4–6). The C-terminal catalytic domain exhibits a canonical α/β-hydrolase fold, characterized by a core structure comprising eight β-strands and several α-helices, which together form a hydrophobic substrate-binding pocket. This domain is equipped with a catalytic triad consisting of serine, histidine, and aspartate (Ser-His-Asp), as well as an oxyanion hole (5, 7). Research has identified two asparagine residues (N78 and N282) within the human AADAC protein sequence, both positioned at potential glycosylation sites. The removal of glycosylation at the N282 site results in a marked reduction in enzymatic activity and substrate binding capacity (4). Furthermore, owing to the structural resemblance of its active site to that of hormone-sensitive lipase (HSL), AADAC has been recently reclassified as a lipase (2, 8). In contrast to carboxylesterases (CES), AADAC demonstrates substrate specificity, with its hydrophobic substrate-binding cavity preferentially accommodating short-chain acyl substrates (e.g., acetyl groups) through spatial constraints, hydrophobic gradient distribution, and stereochemical rigidity constraints (6). Mutations in the catalytic triad (e.g., Ser-217, His-438) or substrate-binding regions (e.g., Phe-190) can significantly impair enzymatic activity or modify metabolic preferences. These properties underscore the potential for developing personalized medicine and targeted therapeutic strategies. Schematic Diagram of AADAC Structure (**Figure 1**).

## 3 Drug metabolism: roles and variability of AADAC

### 3.1 The roles of AADAC in drug metabolism

AADAC, a serine hydrolase, serves as a pivotal enzyme in drug metabolism, activation, and detoxification, playing an essential role in the safe administration of ester drugs (9). It exhibits high expression levels in the liver and gastrointestinal tract, moderate expression in the bladder, and minimal expression in lung and kidney microsomes, with significant expression observed in certain brain regions (10, 11). This distribution facilitates the design of tissue-targeted prodrugs. AADAC is capable of hydrolyzing a wide array of substrates, including rifampicin, a first-line anti-tuberculosis drug associated with multidrug resistance (12); phenacetin, a withdrawn analgesic known to cause methemoglobinemia through AADAC-mediated hydrolysis (13, 14); ketoconazole, an antifungal agent with rare hepatotoxicity (15); and flutamide, an anti-androgen drug linked to liver damage (16). AADAC demonstrates a preference for small acyl groups, particularly acetylated compounds (6), as seen in its hydrolysis of antiplatelet drugs like vicagrel and prasugrel, as well as sedatives such as nitrazepam (17–19). Although it shares tissue distribution and substrate overlap with CES, AADAC is distinguished by its superior catalytic efficiency and unique substrate preferences (20). Its involvement in phenacetin toxicity and flutamide-induced hepatotoxicity highlights its significance in drug safety. However, prolonged exposure to substrates like rifampicin may lead to resistance, complicating infectious disease management. These insights underscore AADAC’s dual role as both a detoxifying agent and a potential contributor to adverse effects, stressing the importance of personalized strategies in drug development and therapy.

### 3.2 The variability of AADAC

#### 3.2.1 Interindividual and population variability of AADAC

Enzymes demonstrate polymorphism, which is evident not only among individuals but also across species. A study examining AADAC in human lung tissue highlighted significant inter-individual variability in the hydrolytic activity of AADAC with respect to phenacetin (21). Polymorphic alleles of AADAC may contribute to variations in enzyme activity and drug response (22). The AADAC gene sequences can differ among individuals, with some individuals carrying mutations such as point mutations, deletions, or insertions. These genetic variations can modify the amino acid sequence of the AADAC enzyme, thereby influencing its three-dimensional structure and function. For example, a study assessing a novel treatment regimen for drug-susceptible tuberculosis patients in southern Africa found that individuals with the AADAC rs1803155 AA variant (65.4%) exhibited a 10.4% reduction in rifapentine clearance rate (23). Furthermore, Sileshi’s study indicated that rifampicin exposure is potentially influenced by both genotype and gender, as well as dosage. In this investigation, 119 adult Ethiopian patients newly diagnosed with tuberculosis underwent a two-week regimen of rifampicin-based anti-tuberculosis therapy. It was observed that male patients exhibited a higher likelihood of reduced plasma exposure to rifampicin (24). Beyond the individual polymorphism of the AADAC gene, the distribution of polymorphisms in the AADAC enzyme also demonstrates variability across different ethnic and geographical populations.

The AADAC gene and enzyme show polymorphism variations across different ethnic and geographical populations. There are three known alleles: AADAC*1, AADAC*2, and AADAC*3. AADAC*1 is found in European Americans, African Americans, Japanese, and Koreans with a frequency of 39.3% to 47.4%. AADAC*2 is present in the same groups with a frequency of 52.6% to 63.5%, and AADAC*3 is found only in European Americans (1.3%) and African Americans (2.0%) (25). *In vitro* studies by the Shimizu group suggest that the AADAC*3/*3 genotype may lead to reduced enzymatic activity. Notably, the AADAC*2 allele frequency in Peruvians is 99.9%, higher than in the other groups (26). Studies indicate that patients with the rs1803155 (AADAC*2 allele) genotype have lower clearance and higher plasma concentration of rifapentine-like rifampin (RIF), possibly contributing to Peru’s high tuberculosis rates (23, 27). Personalized anti-tuberculosis drug regimens are essential to reduce treatment-related side effects, with pharmacogenomics playing a key role in optimizing drug efficacy and safety for individual patients.

#### 3.2.2 Cross-species polymorphism in AADAC

The polymorphism of AADAC is evident across various species. As previously noted, in humans, AADAC mRNA is predominantly expressed in the liver and gastrointestinal tract, with the bladder exhibiting lower levels of expression. Similarly, in marmosets and cynomolgus monkeys, AADAC mRNA is also highly expressed in the liver and gastrointestinal tract (28–30). In contrast, in rats and mice, the highest expression of AADAC mRNA is observed in the liver, followed by the gastrointestinal tract and kidneys (10, 28, 31). Notably, the expression level of AADAC in rat tissues is approximately 7-fold and 10-fold lower than that in human and mouse tissues, respectively. During the hydrolysis of rifamycin, only human and marmoset liver microsomes demonstrate the ability to hydrolyze rifamycin, whereas liver microsomes from rats, mice, dogs, and cynomolgus monkeys exhibit minimal hydrolytic activity. Among rifamycins, only rifabutin is hydrolyzed by marmoset tissue microsomes and recombinant AADAC. Furthermore, the activity of all substrates in marmoset intestinal microsomes is lower than that in liver microsomes (10). These findings underscore the significant interspecies differences in AADAC. Consequently, when assessing the safety and pharmacokinetics of preclinical drugs, it is imperative to carefully select appropriate animal models.

The polymorphic characteristics of AADAC play a crucial role in both the clinical application of pharmaceuticals and the design of scientific research, particularly in the selection of experimental subjects and methodologies. AADAC has been long established as a pivotal enzyme in the hydrolysis of ketoconazole (KC). However, research has demonstrated that AADAC produces varying outcomes in the hydrolytic metabolism of KC. In a study by Fukami, an adenovirus expression system was employed to overexpress AADAC in HepaRG cells, resulting in KC-induced cytotoxicity. In contrast, the application of the AADAC inhibitor, diisopropyl fluorophosphate, alleviated the toxic effects of KC on primary human hepatocytes (32). Meanwhile, research conducted by the Nagaoka team revealed that in Aadac gene knockout mice, the absence of Aadac exacerbated KC-induced liver injury by inhibiting glucocorticoid synthesis and enhancing the inflammatory response (33). The divergent findings can likely be attributed to the differing experimental methodologies employed by the two research groups. Fukami’s cell-based study concentrated on the direct hydrolytic activity of AADAC on KC, wherein the hepatotoxic metabolites produced by KC compromised cellular integrity and function, leading to cytotoxic effects. Conversely, Nagaoka’s *in vivo* study using mice presented a more complex scenario, influenced not only by AADAC hydrolysis but also by additional factors such as glucocorticoids.

## 4 Lipid metabolism: roles of AADAC

### 4.1 Lipolytic activity of AADAC in lipid metabolism

**Figure 2:**
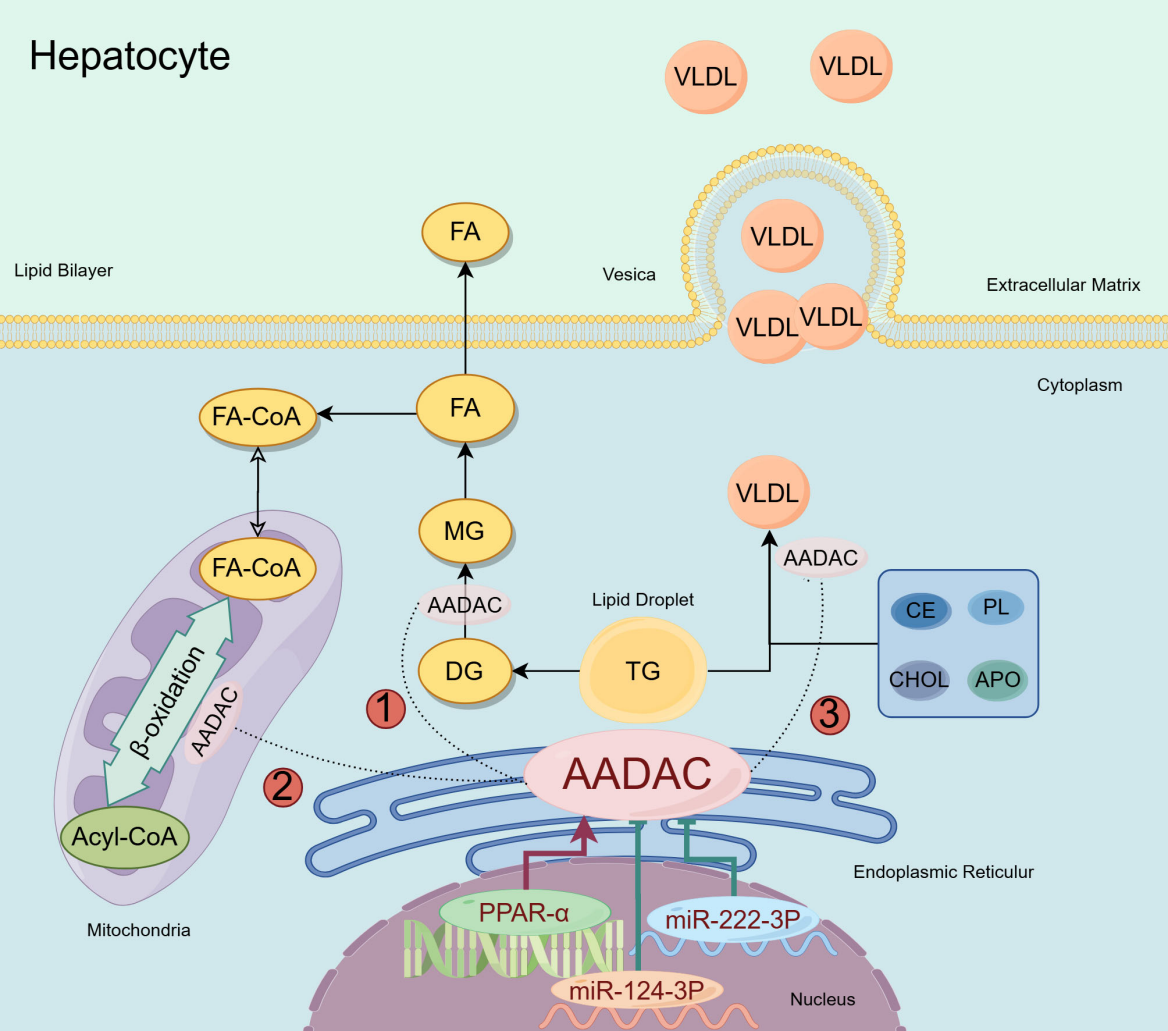
AADAC involved in lipid metabolism in hepatocyte. AADAC is an enzyme in the endoplasmic reticulum that may contribute to lipid metabolism in three ways: ① AADAC is capable of hydrolyzing DG, thereby mitigating the intracellular accumulation of TG; ②AADAC might aid mitochondrial β-oxidation by enhancing TG hydrolysis; ③AADAC facilitates the assembly of VLDL and its subsequent secretion through various mechanisms. The upstream regulatory mechanisms of AADAC: PPAR-α enhances the expression of AADAC, whereas miR-222-3P and miR-124-3P act to suppress its expression.

AADAC is a serine hydrolase localized on the endoplasmic reticulum membrane and is classified within the “GDXG” lipase family. It primarily catalyzes the hydrolysis of cholesterol esters (CE) and triglycerides (TG) (34). Its active site exhibits homology with that of HSL. Numerous studies have demonstrated a significant association between the AADAC gene and lipid metabolism (35, 36). Evidence indicates that stable transfection of HepG2 cells, a human hepatocellular carcinoma cell line deficient in endogenous AADAC, with AADAC results in a 2- to 3-fold increase in endogenous TG secretion (37). In rat hepatoma McArdle RH-7777 cells, which also lack endogenous AADAC, expression of AADAC leads to a reduction in TG accumulation and an enhancement in fatty acid (FA) oxidation (38). Moreover, hepatitis C virus (HCV) infection in Huh7.5 cells results in the downregulation of AADAC expression and defects in lipolysis (39). Reintroduction of AADAC into these infected cells can restore cellular TG lipolysis. AADAC is potentially involved in the regulation of lipid metabolism by exerting its lipase activity, thereby accelerating the hydrolysis of TG into fatty acids and glycerol, which consequently reduces TG accumulation within the cell. The catabolism of fatty acids (FAs) within the cell is further facilitated by the presence of AADAC, which may activate or enhance the pathways involved in intracellular FA oxidation, thereby augmenting FA oxidation. This process leads to the consumption of intracellular FAs, subsequently reducing the substrates available for TG resynthesis and indirectly diminishing TG accumulation. Concurrently, AADAC may modulate proteins or signaling pathways implicated in TG assembly and secretion. It is posited that AADAC may facilitate the assembly of VLDL, given that VLDL serves as the primary vehicle for hepatic TG secretion. By modulating the intracellular lipid milieu, AADAC promotes the incorporation of TG into VLDL, thereby markedly enhancing the secretion of endogenous TG. Furthermore, AADAC may exert an indirect influence on lipid metabolism through the modulation of the endoplasmic reticulum (ER) stress response (40). Under conditions of ER homeostatic imbalance, alterations in AADAC expression may mitigate lipotoxic stress by regulating lipolysis and lipid droplet formation. However, *in vivo* evidence elucidating the role of AADAC in lipid metabolism remains insufficient (**Figure 2**).

### 4.2 Upstream regulation of AADAC in lipid homeostasis

The regulatory role of AADAC in lipid metabolism is not fully understood, but it is known to be controlled by the transcription factor Foxa1. In an experiment using a monoclonal antibody (14 mAb), Foxa1 was found to enhance AADAC expression by binding to a specific promoter region, reducing lipid droplet accumulation in liver cells. This involves the FcRn/PKCδ/Foxa1 pathway, where 14 mAb boosts Foxa1 activity by inhibiting PKCδ phosphorylation, thereby promoting AADAC-mediated lipolysis. Knocking down AADAC or Foxa1 reverses the reduction in lipid droplets, highlighting their regulatory importance. Additionally, studies by Kersten and Richert showed increased AADAC mRNA in human liver cells and mouse hepatocytes treated with peroxisome proliferators (41, 42). Peroxisome proliferator-activated receptor alpha (PPAR-α) plays a crucial role in modulating liver peroxidase activity and the transcription of genes associated with peroxisome proliferation, thereby influencing lipid metabolism (43, 44). Trickett’s research indicates that the expression of Aadac in mouse liver is subject to regulation by circadian rhythms, potentially mediated by PPAR (8). Morikawa’s studies utilizing luciferase and chromatin immunoprecipitation assays have demonstrated that human AADAC expression is regulated by PPAR-α through its interaction with the DR1 (direct repeat sequence 1) located in the -133/-181 region of the AADAC promoter (45). Furthermore, PPAR-α ligands have been shown to significantly elevate AADAC mRNA and protein levels in human hepatoma Hh-7 cells (45). Manipulating PPAR-α expression through knockdown or overexpression results in corresponding decreases or increases in AADAC expression. Collectively, these findings substantiate the conclusion that AADAC expression is governed by the transcription factor PPAR-α.

Research has demonstrated that microRNAs (miRNAs) play a crucial role in regulating lipid homeostasis in the liver, encompassing processes such as fatty acid oxidation, lipid biosynthesis, and lipid excretion (46, 47). Through computational analysis, Sakai identified two potential miR-222-3p recognition elements within the 3’-untranslated region (UTR) of the AADAC gene. Subsequent luciferase assays confirmed that the miR-222-3p recognition element functions to downregulate AADAC expression. Overexpression of miR-222-3p resulted in decreased AADAC activity and expression levels in HEK293 cells, as well as in HepaSH and Huh-1 cells expressing the AADAC protein (48). Furthermore, miR-124-3p has been identified as a regulatory molecule with potential upstream effects on lipid metabolism (49, 50). MiR-124-3p is capable of downregulating AADAC expression and modulating fatty acid and triglyceride homeostasis. Notably, miR-124 also inhibits the replication of the hepatitis C virus (51).

The regulatory mechanisms governing AADAC/Aadac expression remain insufficiently elucidated. Conversely, evidence suggests that Aadac expression in the murine liver is modulated by adrenergic receptors (AR) (52). The AR-related signaling pathway constitutes a significant element of the stress response system. Activation of AR leads to the upregulation of the Aadac gene. Notably, AR activation concurrently results in the inactivation of the hepatic insulin/PI3K/AKT/FoxO1 signaling pathway, which is integral to the regulation of genes implicated in triglyceride synthesis. Furthermore, the interaction between immune cells and hepatocytes significantly influences the regulation of AADAC. Interleukin-10 (IL-10) exerts a negative regulatory effect on AADAC expression (53). In wild-type (WT) mice, the expression of Aadac in the intestine is mediated by the signal transducer and activator of transcription 3 (STAT3), rather than by nuclear factor kappa B (NF-κB). IL-10 may modulate Aadac expression through its action on the STAT3 signaling pathway. It is hypothesized that IL-10 reduces the expression and activity of AADAC by inhibiting the transcription of the AADAC gene or by decreasing its protein stability. This reduction may lead to lipid metabolic disorders, potentially playing a critical role in the pathogenesis of diseases such as non-alcoholic fatty liver disease.

## 5 Activators and inhibitors of AADAC

Recent studies on AADAC agonists are limited. AADAC is regulated by transcription factors, miRNAs, and inflammatory factors, and has been identified as a cardioprotective agent in type 2 diabetes (T2D) patients, aiding in cardiovascular health and lipid balance (54). Developing AADAC activators could advance treatments for cardiovascular diseases in T2D patients. No specific AADAC inhibitor exists yet, but simvastatin and mevastatin are known to inhibit its activity (55, 56). Vincristine and physostigmine, as AADAC inhibitors, also inhibit the CES2 enzyme, potentially impacting AADAC research (9, 13, 57). Epicatechin gallate and epigallocatechin gallate have shown promising AADAC inhibition, suggesting potential for further drug development (58). Developing AADAC inhibitors faces several challenges: significant species differences complicate safety and pharmacokinetic evaluations, necessitating careful animal model selection. Variations in AADAC protein expression and gene polymorphisms affect hydrolytic activity, making it hard to predict individual patient responses. Additionally, limited research on AADAC compared to other esterases hinders inhibitor development.

## 6 Effect of AADAC on metabolic diseases

### 6.1 AADAC in obesity

Researchers from the National University of Singapore, in collaboration with colleagues from the United States and China, conducted a study utilizing metabolic disease data from the Global Burden of Disease Report spanning the years 2000 to 2019. Their analysis revealed that obesity exerted the most significant and consistently increasing influence on mortality among metabolic diseases (59). AADAC, functioning as a lipase, plays a critical role in the lipolysis of TG, facilitates FA oxidation, and impacts the assembly of VLDL in hepatocytes, as well as the activation and re-esterification of TG within the cytosolic pool. The onset of obesity is intricately linked to dysregulated fat metabolism, characterized by diminished fat breakdown and enhanced fat accumulation. The role of AADAC in TG metabolism may indirectly affect the equilibrium between fat storage and utilization in the body, thereby associating it with obesity. For instance, alterations in AADAC activity could lead to disruptions in TG decomposition or re-esterification processes, thereby influencing fat metabolism and subsequently affecting the onset and progression of obesity. Clinical data indicate that obese individuals exhibit reduced hepatic expression of AADAC compared to their lean counterparts (60). This observation suggests that the expression level of AADAC plays a significant role in lipid metabolism. The down-regulation of AADAC may result in a decreased rate of TG lipolysis, thereby impairing the normal process of VLDL assembly and FA β-oxidation. This disruption can lead to lipid metabolism disorders and contribute to obesity. It is conceivable that enhancing the expression of AADAC could augment lipase activity, thereby ameliorating disordered lipid metabolism in obese patients. Furthermore, obesity is a major risk factor for the development of diabetes and coronary heart disease.

### 6.2 AADAC in diabetes

Type 2 diabetes (T2D) is rapidly increasing worldwide, becoming a major 21st-century health crisis alongside obesity (61). There is a strong link between lipid metabolism and T2D, with dyslipidemia common in T2D patients. Disrupted lipid metabolism leads to excess free fatty acids (FFAs) in the blood, impairing insulin signaling and causing insulin resistance—a key factor in T2D development. Adipokines like adiponectin and leptin, secreted by fat tissue, are crucial for regulating lipid metabolism and insulin sensitivity. In T2D patients, adiponectin levels are typically reduced, hindering insulin sensitivity and FA oxidation, and disrupting normal lipid and glucose regulation. In the context of insulin resistance, the physiological roles of insulin in facilitating lipogenesis and inhibiting lipolysis are compromised. Consequently, there is an increased release of FFAs into the bloodstream, which further aggravates dyslipidemia. Chronic disturbances in lipid metabolism, characterized by elevated concentrations of FFAs and TGs in the circulation, can induce lipotoxicity in pancreatic β-cells, thereby impairing their functionality. This impairment leads to a reduction in insulin secretion, which is insufficient to meet the body’s requirements for glycemic control, thereby exacerbating hyperglycemia and contributing to the pathogenesis of type 2 diabetes mellitus.

### 6.3 AADAC in cardiovascular diseases

In fact, there is a strong association among cardiovascular diseases, obesity, and diabetes (62). Diabetes-induced hyperlipidemia is a key risk factor for diabetic macroangiopathy, with metabolic syndrome elements like diabetic dyslipidemia, hyperglycemia, and insulin resistance speeding up atherosclerosis (63–65). Atherosclerosis is the main pathological basis of ischemic cardio-cerebrovascular diseases such as coronary heart disease and cerebrovascular disease, and its pathogenesis is closely related to prognosis (66, 67). The occurrence and development of Atherosclerosis are closely related to lipid accumulation and inflammation (68). Research demonstrated an inverse correlation between AADAC expression and the magnitude of atherosclerotic plaques (69). Increased AADAC levels in vascular smooth muscle cells (VSMCs) of T2D patients reduce lipid accumulation, cell proliferation, migration, and possibly cell death, lowering atherosclerosis risk and protecting against heart disease (70). The research team led by Pang, while exploring the potential molecular mechanisms of supraventricular tachycardia (SVT), found that the expression of the AADAC gene was decreased in SVT samples, showing significant potential for clinical diagnosis (71). Moreover, research suggests the AADAC gene could be a biomarker for ruptured intracranial aneurysms, offering a novel target and treatment approach (72). The cardiovascular disease is one of the leading causes of death worldwide, and its prevalence continues to rise. As a new therapeutic target and biomarker, AADAC may play an important role in the diagnosis and prognosis of cardiovascular diseases by participating in lipid metabolism and affecting diabetes.

### 6.4 AADAC in cancer

#### 6.4.1 AADAC in digestive system cancer

In the Global Cancer Statistics 2020, it was found that the incidence and mortality of digestive system cancers still ranked among the top in the world (73). Recent studies have shown that AADAC has great potential in the treatment and prognosis of cancer. Compared with normal tissues, the expression of AADAC is downregulated in esophageal cancer. AADAC is not only related to the prognosis of patients with esophageal squamous cell carcinoma (74), but also a potential new diagnostic and prognostic biomarker for the development of Barrett’s esophagus (BE) into esophageal adenocarcinoma (EAC) (75). In a study by Wang investigating the regulatory effect of circular RNA hsa_circ_0043603 on the progression of esophageal squamous cell carcinoma (ESCC), it was confirmed that AADAC is a target of miR - 1178 - 3p. Hsa_circ_0043603 is a circular RNA that regulates the overexpression of AADAC by interacting with miR - 1178 - 3p, thereby inhibiting the growth and spread of esophageal squamous cell carcinoma (76).

AADAC expression in gastric cancer tissues is inconsistent. The GEPIA database indicates significantly lower AADAC expression in cancerous tissues compared to non-cancerous ones (77). In contrast, Abdel-Tawab’s study found AADAC upregulated in gastric cancer, correlating with tumor grade and stage (78). The observed discrepancy may be attributed to variations in sample size. In contrast to the GEPIA database, the study conducted by Abdel-Tawab included only 40 samples, comprising 20 gastric cancer tissue samples and 20 control samples. Furthermore, the gastric cancer tissue samples were derived from tumors at various stages and grades. Tumor heterogeneity could be a factor, as tumors contain various sub-clones. Cancer cells in different areas may exhibit diverse gene expression due to genomic instability. Additionally, the same cancer type can show different gene expression patterns across patients due to genetic background, environmental factors, or epigenetics. In addition, AADAC is an independent prognostic factor for Borrmann type III advanced gastric cancer, with high expression linked to improved overall and disease-free survival (79). It may serve as a new therapeutic target and aids in guiding treatment strategies for gastric cancer (80–83).

The expression of AADAC is upregulated in pancreatic cancer, as reported by Wu et al., who propose that AADAC may contribute to chemotherapy resistance and possess prognostic significance (84). A predictive model, derived from clinical and genetic data, could facilitate the assessment and management of treatment for patients with resectable pancreatic cancer. Ferroptosis, a recently characterized process, involves the accumulation of iron-dependent lipid peroxides to toxic levels, resulting in cell death (85, 86). In liver metastases of colon cancer, elevated AADAC levels promote cellular proliferation and inhibit lipid peroxidation by activating NRF2 and upregulating SLC7A11 expression, thus protecting metastatic cells from iron deficiency (87).

#### 6.4.2 AADAC in reproductive system cancer

AADAC is implicated in the pathogenesis of reproductive system cancers, with its expression notably upregulated in ovarian cancer tissues. The overexpression of AADAC has been shown to inhibit the malignant progression of ovarian cancer cells. Both cisplatin and imatinib are known to suppress the malignant progression of cancer cells, and the overexpression of AADAC synergistically enhances this inhibitory effect (88). Zhao et al. have identified AADAC as a potential N1-methyladenosine-related biomarker for the prognosis of ovarian cancer (89). High expression levels of AADAC are significantly and independently associated with improved survival outcomes in ovarian cancer patients. Furthermore, the upregulation of AADAC is positively correlated with the infiltration of CD4+ memory T cells. The expression profile of AADAC demonstrates a significant and independent association with both the survival outcomes of ovarian cancer patients and the infiltration of CD4+ memory T cells, suggesting its potential utility in predicting prognosis and the efficacy of immunotherapy (90, 91). These findings offer novel insights and potential biomarkers for the treatment of ovarian cancer.

The function of AADAC in cancer exhibits variability across different populations: it is oncogenic in European and American populations, whereas it functions as an anti-oncogene in Asian populations (92). Although the role of AADAC in cancer is not yet fully elucidated, its expression levels may serve as indicators of cancer prognosis. Further research is required to effectively integrate AADAC into cancer prognostic evaluations (**Table 1**).

**Table 1:** Expression changes and effects of AADAC in diseases.

Diseases		The Expression Changes	Effects on Diseases	Reference
Cardiovascular Diseases	Atherosclerosis *	/	Protective factor	Misra & Fisher, 2020; Toyohara et al., 2020 (69, 70)
	Intracranial Aneurysms	Up	Risk factor	Li et al., 2022 (72)
Cancer	Esophageal Carcinoma Gastric Cancer	Down Up Down	Protective factor Protective factor Protective factor	X. Wang et al., 2023; Yi et al., 2023 (75, 76) Abdel-Tawab et al., 2022 (78) Liu et al., 2018 (83)
	Pancreatic Cancer Colorectal Cancer Ovarian Cancer	Up Up Up	Protective factor Risk factor Protective factor	C. Wu et al., 2020 (84) Sun et al., 2022 (87) H. Wang et al., 2022 (88)

The table mainly summarizes the changes and effects of AADAC expression levels in different diseases.

## 7 Effect of AADAC on neurological diseases

AADAC is a hydrolase found in the liver, intestinal mucosa, and brain of mammals, primarily catalyzing the deacetylation of arylamine compounds. It may affect neurotransmitters like dopamine and serotonin, influencing synaptic transmission and neural signaling. Research has shown AADAC can convert N-acetylserotonin (NAS) into serotonin, supporting its role in neurotransmitter metabolism (93). Although the specific mechanisms remain unexplored, AADAC is also linked to drug metabolism(such as antiepileptic drugs and hypnotic - sedative drugs), potentially impacting the distribution and activity of drugs in the nervous system through deacetylation (19, 22, 57). The metabolism of certain brain drugs relies on AADAC activity, and gene polymorphisms can influence drug response. The role of AADAC in lipid metabolism may impact neuronal membrane integrity and synaptic function. Specific AADAC gene single nucleotide polymorphisms (SNPs) are linked to disease risk, with studies suggesting AADAC as a factor in Tourette syndrome (11, 94–96). Vadgama et al. found a paternal stop-loss variant in AADAC in twins with Tourette syndrome, indicating that genetic variations in AADAC may heighten susceptibility to nerve damage (97).

Research on AADAC in neuroscience is still in its early stages, and its mechanisms need further investigation. Future studies should focus on AADAC’s catalytic effects on neurotransmitters like dopamine and acetylcholine through *in vitro* experiments, and assess its impact on nervous system phenotypes using animal models with AADAC gene knockout or overexpression.

## 8 Discussion

AADAC, a member of the polygenic hydrolase family, is a microsomal serine esterase predominantly located in the liver and intestine. It plays a pivotal role in drug and lipid metabolism, as well as in the pathogenesis and progression of various diseases. In the context of drug metabolism, AADAC plays a crucial role in the metabolism, activation, and detoxification of drugs, which is highly significant for the personalized and safe use of ester drugs. AADAC exhibits substrate specificity, with a preference for short-chain acyl substrates. Gene polymorphism of AADAC is prominent among individuals and species, influencing enzyme activity, drug response, clinical drug application, and the design of scientific research experiments. In terms of lipid metabolism, AADAC possesses lipase activity, catalyzing the hydrolysis of cholesterol esters and TGs, thereby participating in lipid mobilization and affecting FA oxidation, VLDL assembly, and the metabolic balance of TGs. Its expression is regulated by various factors, including transcription factors (such as Foxa1 and PPAR-α), microRNAs (such as miR-222-3p and miR-124-3p), inflammatory factors (such as IL-10), and adrenergic receptors.

AADAC is intricately associated with various metabolic diseases. In individuals with obesity, hepatic expression of AADAC is reduced, and alterations in its activity may influence triglyceride metabolism, thereby contributing to the onset and progression of obesity. In the context of diabetes, there is a reciprocal interaction between lipid metabolism disorders and T2D, with AADAC potentially playing an indirect role. In terms of cardiovascular diseases, the expression of AADAC is negatively correlated with the size of atherosclerotic plaques, and it has potential value in the diagnosis of supraventricular tachycardia and intracranial aneurysms. In oncology, the role of AADAC is multifaceted; its expression is diminished in esophageal and certain gastric cancers, correlating with patient prognosis, whereas it is elevated in pancreatic and ovarian cancers. Notably, in ovarian cancer, AADAC overexpression can inhibit malignant cell progression and exhibit a synergistic anti-tumor effect when combined with chemotherapeutic agents. Furthermore, the role of AADAC in cancer exhibits variability across different populations. Within the nervous system, AADAC is implicated in the regulation of neurotransmitter metabolic equilibrium and the metabolism of pharmaceuticals, thereby influencing neural signal transduction and maintaining the integrity of neuronal cell membranes. Additionally, single-nucleotide polymorphisms in the AADAC gene have been linked to disorders such as Tourette syndrome. At present, there is a limited body of research concerning AADAC agonists. AADAC is closely associated with metabolic diseases and holds promise as both a biomarker and a potential therapeutic target. Future studies should prioritize elucidating its tissue-specific regulatory mechanisms across diverse metabolic contexts, while advancing the development of selective AADAC agonists and inhibitors to enhance its precision medicine applications in disease diagnosis and treatment.
